# Cardio Protection and Antiatherosclerotic Effects of *Solanum incanum* (Lin.) Extracts in Animal Models

**DOI:** 10.1155/sci5/5594777

**Published:** 2025-03-29

**Authors:** Stephen Ngigi Mburu, Mathew Piero Ngugi, John K. Mwonjoria

**Affiliations:** ^1^Department of Medical Laboratory Sciences, Kenyatta University, Nairobi, Kenya; ^2^Department of Biochemistry, Microbiology and Biotechnology, Kenyatta University, Nairobi, Kenya

**Keywords:** cardiology, cardio protection, cardiovascular, coronary vascular disease, hyperlipidemia, *Solanum incanum*

## Abstract

*Solanum incanum* is a bushy perennial herb which is used for treatment of various ailments in East Africa including stomach pain, toothaches, ulcers, and cardiovascular diseases. However, there is scarcity of data on its safety, and its effect on the physiology of the cardiovascular system. Hence, this study envisaged evaluating the effect of methanol extract of the herb on myocardial action atherosclerotic tendency and safety. Myocardial activity assay involved determination of the heart rate and force of contraction using an isolated rabbit heart in an organ bath and kymograph, while antiatherosclerotic effects were assayed on blood obtained from white Wistar rats fed on high lipid diet where the levels of low-density lipoproteins, high-density lipoproteins and total cholesterol were determined. Assays of the toxic effects of the extract were carried out on Swiss albino mice while qualitative phytochemical analyses were carried out using one-way Anova and *Tukey* as the *post hoc* test, a value of (*p* < 0.05) was taken as the limit of significance. The plant extracts exhibited an increase in the force of contraction of the heart and decrease in heart rate. It also caused significant (*p* < 0.05) reduction in serum low-density lipoproteins and total cholesterol as well as elevation of HDL but no effect on hematological parameters. Phytochemical analyses showed the presence of alkaloids, glycosides, saponins flavonoids, terpenoids, steroids, and phenolics. Hence, the *S. incanum* root extract may contain compounds with antiatherosclerotic effects that are cardioprotective and therefore a potential source of novel remedies for the ailment.

## 1. Introduction

Since time immemorial plant extracts have been shown to be effective in the management of various illnesses. *Solanum incanum*, often called Sodom/bitter apple-English, *Muturere or Mutongu*-Kikuyu, *Entulele*-Maasai. Roots and leaves are edible and are used as soup flavor [1]. *Solanum incanum* extracts possess antimicrobial [2], antihyperlipidemic, antidiabetic [3], anticarcinogenic [4], antifungal [5], antischistosomal [6], cytotoxic [5], anti-inflammation, antinociceptive [7, 8], and antinociceptive wound healing [1, 7] activities. Several phytochemicals isolated from *S. incanum* includes, alkaloids, resins, glycosides, flavonoids, steroids, solasodine, triterpenes, and phenolics [1]. There is limited reported studies on the cardioprotective and antiatherosclerotic effects. Knowledge about their safety is also scanty. This study aimed in evaluating antiatherosclerotic, antihyperlipidemic and cardioprotective effects of *Solanum incanum* in laboratory animal models.

## 2. Methodology

### 2.1. Plant Materials Preparation

Roots of *Solanum incanum* were gathered from Nova pioneer, Kamiti region in Kiambu County. After being cleaned, sliced, and shielded from the sun, they were dried. For three and a half weeks, this was carried out in Kenyatta University's biological chemistry lab. Taxonomist verification came next, and the university herbarium's botanical exhibit number (SM2022/02TB) was assigned. They were then ground by electric grinding mill into powder weighed and stored at room temperature for extraction.

### 2.2. Plant Materials Extraction

A weighing scale equipment was used to measure 100 g of the plant powder. It was soaked in 250 mL of methanol on repetition for 72 hours. The mixture was stirred for 8 seconds to formulate mixing. It was then allowed to stand for 2 h, decanted and addition of more methanol was done and allowed stand for 24 h. For a duration of as 72 h, this was done repeatedly. After being decanted, the supernatant was filtered using Whatman No. 1 filter paper. To obtain the extract, the filtrate solution was concentrated to 1 mg/mL using a rotor evaporator at a lower pressure. The extract was subsequently stored in airtight bottles until it was needed again.

### 2.3. Experimental Animal Models

Male White New Zealander for cardiotonic examinations, four to five-month-old rabbits weighing 1000g were chosen. For toxicity investigations, 10 Swiss Albino mice, weighing between 18 and 24 g and evenly distributed, both male and female, aged six to eight weeks, were used. Hyperlipidemic investigations were conducted using 30 male and female Wistar rats weighing about 50 and 60 g, aged eight to 10 weeks. Before the experiment began, they were placed in cages at room temperature and given 7 days to acclimate. Access to food and tap water was offered *ad lipitum*.

Experiments were conducted as per the guidelines for laboratory animal use and care [9], whereas the National Commission for Science and Technology (NACOSTI) and the Kenyatta University Animal Care and Use Committee approved the research authorization.

### 2.4. Drugs and Chemicals

Drugs and chemicals used are: petroleum normal saline, methanol, ether, formalin, drug statin (avastatin), Dimethyl sulfoxide, Tyrode solution (NaCl 8.0 g/L, KCl 0.28 g/L, CaCl 0.2 g/L, MgCl2 0.1 g/L, NaH2PO4 0.05 g/L, NaHCO3 1.0 g/L, and glucose 1.0 g/L), distilled water adrenaline, acetylcholine, atropine, and propylthiouracil.

### 2.5. Bioassays

#### 2.5.1. Cardiotonic Assays

The Langendorff method [10] was used to perform the cardiotonic experiment. On the 29^th^ day of the experiment, the rabbits were euthanized with chloroform. A skilled anatomical technologist opened the chest right away. Just below the aortic bifurcation, the heart and aorta were removed. After that, it was kept at 37°C in a warm, aerated Tyrode solution. The heart was placed in the organ bath with Tyrode attached to a kymograph that had a recorder and a revolving drum. Acetylcholine (0.5 mg/mL), adrenaline (0.5 mg/mL), and plant extracts (25, 50, and 100 mg/kg) were the doses given. The isolated rabbit heart was administered with the dosages of the plant extracts.

#### 2.5.2. Lipidemic Assay

The methodologies provided by [11] were used to perform lipidemic experiments. Lipidemic experiments were conducted on male and female Wistar rats weighing 50–60 g at 8–10 weeks of age. They were fed a diet heavy in fat and cholesterol. 10% York egg (5.6 g/bw), 10% lard (5.6 g/bw), 0.2% cholic acid (0.112 g/bw), and 0.59% propylthiouracil (0.28 g/bw) comprised the high-cholesterol, high-fat diet. They were divided into five-person groups (*n* = 5). *Solanum incanum* extract at doses of 25, 50, and 100 mg/kg was administered to each group. The fifth group received distilled water (1 mL) orally every day for 28 days, while the fourth group received 40 mg/kg of avastatin. The medications were administered orally every day by gavage. Animals were monitored for any behavior changes which could arise due to toxicity such as vomiting, diarrhea, ataxia, or even death. Body weights were recorded on a weekly basis. On the 28^th^ day, the animals were euthanized by cotton applied with chloroform and blood sample collected through a cardiac puncture. Weights of the liver, kidney, spleen, lungs, heart, and brain were also recorded.

#### 2.5.3. Toxicity Assay

In the subacute toxicity investigation, male and female albino mice weighing between 18 and 25 g on average were aged between 6 and 7 weeks. Before the trial started, they spent a week getting used to the cages at room temperature. There was an unlimited supply of standard commercial rat pellets available. Animals were divided into three groups after a week of acclimatization, and they were given 100, 170, and 300 mg/kg bw of *S. incanum* extracts, respectively. Schaeffer's formulas were used for this. The corresponding doses were high, medium, and low. The baseline, experimental group four, was given distilled water. The techniques outlined by Somade. et al. [12] were used to determine the dosage levels.

After experimentation, animals were sacrificed and blood was collected through the cardiac puncture for biochemical analysis. Quality control and calibration of the analyzer was done prior to testing.

### 2.6. Phytochemical Screening

Qualitative phytochemical testing was conducted using the techniques outlined by Shaikh and Patil (2020).

### 2.7. Statistical Analysis

Tukey's post hoc test and one-way ANOVA were used to evaluate the data, which were presented as means and standard error of the means. The threshold for significance was set at *p* < 0.05.

## 3. Results

Means and SEM that do not share a lowercase letter along the column, as well as means of percentage change in heart rate (within parenthesis) that does not share a letter along the column are, significantly different. Means that do not share an uppercase superscript letter along the row, are significantly different (*p* < 0.05).

## 4. Discussion

**Figure 1:**
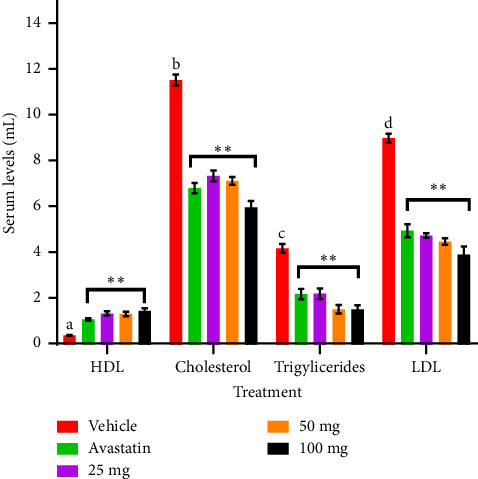
Effects of *Solanum incanum* extracts on high fat diet induced hyperlipidemia in rats. Bars with ⁣^∗∗^Superscript is significantly different *p* < 0.001 from the rest with the same parameters.

In this study, the doses of *Solanum incanum* extract significantly (*p* < 0.05) reduced serum levels of low-density lipoprotein and total serum cholesterol with the effect of the 100 mg/kg dose being significantly higher than the standard drug used (Table 1). However, all the treatments caused significant elevation of the high-density lipoprotein and as observed before the 100 mg/kg doing better than avastatin though in both cases, the effects were not doses dependent. Elevated levels of blood lipids i.e., low-density lipoproteins, cholesterol and triglycerides, as well as reduced levels of high-density lipoproteins results in a disorder known as hyperlipidemia [13] which is a major cause of coronary artery diseases (CAD) and ultimately congestive heart failure [14]. The underlying cause of CAD is usually atherosclerosis, a condition characterized by the buildup of fats which in turn causes atherosclerotic plaques. These are composed of cholesterol, fatty substances, cellular waste products, calcium, and fibrin on the inner walls of arteries. There was significant elevation of fats and a total increase of body weights in rats characterized by hyperlipidemia (Table 2). Various experimental groups portrayed unique phenomena in relations to doses given, and the plant extracts (Table 3). These plaques restrict blood flow to the myocardium. [15]. Reduction of LDL levels and increase of high-density lipoprotein reduce the risk of cardiovascular disorders. This varied in various doses and standard drug used (Figure 1). Factors such as various remedies and diets that aid in reduction of hypercholesterolemia can play a key role in its alleviation [16]. The effects observed in this study shows that the *S. incanum* root extracts may be beneficial in management of atheromatous plaque and resultant cardiovascular diseases. Similar activities have been reported with related plants like *Solanum melongena* [16, 17] *Solanum torvum* [18] though study by Praca, Andrea, and Carameli [17] showed that *Solanum melongena* infusion with orange juice had a modest and transitory effect on hypercholesterolemia in human subjects hence could not be recommended as an alternative remedy to statins. Antihyperlipidemia drugs such as avastatin, fibrates, atorvastatin, fluvastatin, and lovastatin are often used to treat hyperlipidemia [20, 21]. The cardioprotective and antiatherosclerotic effects of *Solanum incanum* are attributed to its bioactive compounds, including flavonoids, saponins, and terpenoids, which act through multiple molecular mechanisms. Flavonoids inhibit HMG-CoA reductase, reducing cholesterol synthesis, and upregulate ABCG5/ABCG8 transporters to enhance cholesterol excretion [22, 23]. Saponins lower lipid levels by binding intestinal cholesterol and increasing CYP7A1 expression, promoting bile acid synthesis [24]. Terpenoids exert anti-inflammatory effects via NF-κB inhibition, reducing cytokine production, and enhance eNOS activity, improving vascular function [25]. Additionally, flavonoids and terpenoids activate PPAR-α/γ pathways, increasing fatty acid oxidation and reducing foam cell formation, thus preventing atherosclerosis [26, 27]. These mechanisms collectively contribute to the lipid-lowering, anti-inflammatory, and endothelial-protective effects of *S. incanum*, supporting its potential as a cardiovascular therapeutic agent. Over the years, medicinal plants have served as essential bio-resources for the management of many diseases and disorders [13, 28]. It may also suffice to say that *S. incanum* extract may contain secondary metabolites that may of great value in synthesis of novel compounds or remedies for CAD.

**Figure 2:**
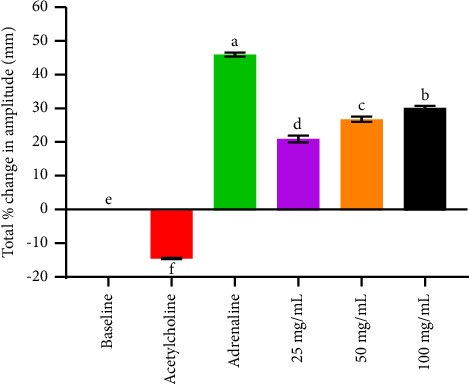
Effect of methanol extract of *Solanum incanum* on total percentage change in height of force of contraction of rabbit's isolated heart. Bars that do not share a letter are statistically significant using one-way ANOVA and Tukey's multiple comparisons (*p* < 0.05).

Cardiotonic medications improve the efficiency and contraction of the heart muscles, thereby improving the tissue perfusion with blood [15]. Extracts of *S*. *incanum* exhibited a significant (*p* < 0.05) decrease in heart rate and increased in force of contraction of the isolated heart (Figure 2), which was comparable to that of adrenaline but was not dose dependent while acetylcholine significantly decreased both the heart rate and force of contraction (Table 4). The two parameters namely the heart rate (chronotropism) and myocardial force of contraction (inotropism) are the key factors that determine the cardiac out which is the numerical product of the duo [29]. The cardiac output (C.O.) that refer to the amount of blood ejected out of the heart per minute is a very important determinant of blood pressure [12, 13]. Any factor that increases the C.O. directly increase blood pressure or hypertension. Being a major cause of morbidity and mortality in the world. The extract had a negative chronotropic effect meaning it would lower the blood pressure however this is thwarted by the negative ionotropic effect (Table 5). Hence, it would not be expected to cause a major shift in C.O. [12, 13]. The positive ionotropic effects that were exhibited by the studied extracts (Table 5) may therefore be attributed to cardiac stimulation or through increased availability of intracellular Ca^2+^ by opening membrane L-type Ca^2+^ channels or via other mechanisms such as inhibition of potassium channels and β1-adrenoceptors. The cardiotonic activities of *S. incanum* extracts can be attributed to the classes of phytocompounds that were identified using qualitative analysis. The extract noted the presence of glycosides, flavonoids, phenolics, saponins, alkaloids, terpenoids and steroids (Table 6). This could explain why *S. incanum* extract had a better cardiotonic effect. The phytochemical classes of flavonoids, steroids, glycosides phenolics and terpenoids have been associated with cardiotonic effects [12, 13, 30, 31]. For instance, glycosides inhibit Na^+^/K^+^ ATPase resulting in increased intracellular Ca^2+^ concentrations via Na^+^/Ca^2+^ exchange. This causes both the transient and slow inward Ca^2+^ influx to rise [32]. Besides, flavonoids (such as kaempferol and quercetin) have also been shown to increase cardiac muscle contraction [15].

Sub-acute toxicity study shows the adverse outcomes after dose exposure of a test drug in a short period. The acute toxicity effects of methanol extract of *S. incanum* at doses of 1000 and 2000 mg/kg did not show any toxicity signs or behaviors after the dose exposure in mice (Tables 7, 8, and 9).

## 5. Conclusion

The methanol extract of *S. incanum* significantly lowered the plasma levels of LDL, and total cholesterol concentration besides elevating the level of HDL in rats therefore it exhibits potential antiatherosclerosis effects. It also exhibited negative chronotropic and positive ionotropic effects with isolated rabbit hearts and hence may not have a profound effect on cardiac output and ultimately the blood pressure. Therapeutic doses of *S. incanum* showed no significant effects on both hematological and biochemical parameters and hence may not be toxic. By altering the LDL:HDL ratio, the extract may be deemed to have exhibited cardio-protective effect. The methanol extracts of *S. incanum* possess classes of phytocompounds associated with cardio-protective effects such as flavonoids and saponins.

## Figures and Tables

**Table 1:** Effect of *S incanum extracts* on lipid profiles following high-fat-diet-induced hyperlipidemia in rats.

Treatment	Lipid profiles (mmol/L)			
	HDL	TC	TRIG	LDL
Baseline	1.63 ± 0.12^b^	1.57 ± 0.11^d^	0.71 ± 0.70^d^	0.41 ± 0.07^d^
Negative control	0.40 ± 0.02^d^	11.57 ± 0.24^a^	4.21 ± 0.19^a^	9.02 ± 0.18^a^
HFD + avastatin	1.11 ± 0.05^c^	6.83 ± 0.22^b^	2.21 ± 0.23^b^	4.98 ± 0.28^b^
HFD + 25 mg	1.44 ± 0.05^bc^	6.31 ± 0.16^b^	1.55 ± 0.10^c^	4.14 ± 0.12^b^
HFD + 50 mg	1.75 ± 0.10^b^	4.40 ± 0.40^c^	1.10 ± 0.05^cd^	3.04 ± 0.43^c^
HFD + 100 mg	2.19 ± 0.12^a^	3.54 ± 0.08^c^	0.94 ± 0.05^d^	2.23 ± 0.26^c^

*Note:* Means and SEM that do not share a letter along the column are significantly different (*p* < 0.05).

**Table 2:** Effect of *S. incanum* extracts on body weights following high-fat-diet-induced in rats.

Treatment	Percentage change in body weights (g)				
	Day 7	Day 14	Day 21	Day 28	Total change
Baseline	11.32 ± 1.23^cD^	30.27 ± 0.59^dC^	54.65 ± 1.06^dB^	68.85 ± 1.25^dA^	41.27 ± 0.63^e^
Negative control	26.52 ± 0.69^aD^	52.49 ± 0.90^aC^	81.61 ± 1.21^aB^	95.34 ± 0.88^aA^	63.99 ± 0.55^a^
Avastatin	21.06 ± 0.72^bD^	42.13 ± 0.94^bC^	62.31 ± 0.88^bcB^	79.98 ± 1.00^cA^	51.37 ± 0.10^c^
25 mg	21.59 ± 0.95^bD^	45.06 ± 0.72^bC^	64.62 ± 1.00^bB^	84.50 ± 0.53^bA^	53.94 ± 0.37^b^
50 mg	21.38 ± 1.31^bD^	38.18 ± 0.58^cC^	60.82 ± 0.64^bcB^	79.61 ± 0.71^cA^	50.00 ± 0.25^cd^
100 mg	19.60 ± 0.86^bD^	36.39 ± 0.58^cC^	59.63 ± 0.88^cB^	76.88 ± 0.90^cA^	48.13 ± 0.46^d^

*Note:* Means and SEM that do not share a letter along the column are significantly different. Means and SEM that do not share an uppercase superscript letter are significantly different (*p* < 0.05).

**Table 3:** Animal model distribution for experimental groups.

Experimental groups	*N* = 5
Baseline (normal animal feeds *ad lipitum*)	5
Negative control (high fat diet)	5
Positive control (avastatin)	5
25 mg/kg	5
50 mg/kg	5
100 mg/kg	5

**Table 4:** Chronotropic effects of *S. incanum* extracts on isolated rabbit's heart.

Treatment	0 min	Heart rate (percentage change in heart rate)				
		1^st^	2^nd^	3^rd^	4^th^	5^th^
Baseline	210.00 ± 2.89^aA^	210.00 ± 2.89^bA^	208.33 ± 1.67^bA^	208.33 ± 1.67^bA^	210.00 ± 2.89^bA^	211.67 ± 1.67^bA^
	(0.00 ± 0.00)	(0.00 ± 0.00^b^)	(0.00 ± 0.00^b^)	(0.00 ± 0.00^b^)	(0.00 ± 0.00^b^)	(0.00 ± 0.00^b^)

Acetylcholine	210.00 ± 2.89^aA^	178.33 ± 1.67^dB^	161.67 ± 1.67^eC^	141.67 ± 1.67^eD^	131.67 ± 1.67^eE^	121.67 ± 1.67^eF^
	(0.00 ± 0.00)	(−15.08 ± 0.79^d^)	(−22.40 ± 0.80^e^)	(−32.00 ± 0.80^e^)	(−37.3 ± 0.79^f^)	(−42.52 ± 0.79^e^)

Adrenaline	211.67 ± 1.67^aD^	230.00 ± 2.89^aC^	245.00 ± 2.89^aB^	250.00 ± 2.89^aAB^	260.00 ± 2.89^aA^	260.00 ± 2.89^aA^
	(0.00 ± 0.00)	(9.52 ± 1.37^a^)	(17.60 ± 1.39^a^)	(20.00 ± 1.39^a^)	(23.81 ± 1.37^a^)	(22.83 ± 1.36^a^)

25 mg/mL	208.33 ± 1.67^aA^	196.67 ± 1.67^cB^	188.33 ± 1.67^cBC^	185.00 ± 2.89^cCD^	176.67 ± 1.67^cDE^	171.67 ± 1.67^cE^
	(0.00 ± 0.00)	(−6.35 ± 0.79^c^)	(−9.60 ± 0.80^c^)	(−11.20 ± 1.39^c^)	(−15.87 ± 0.79^c^)	(−18.90 ± 0.79^c^)

50 mg/mL	210.00 ± 2.89^aA^	193.33 ± 1.67^cB^	181.67 ± 1.67^cdC^	171.67 ± 1.67^dD^	161.67 ± 1.67^dDE^	156.67 ± 1.67^dE^
	(0.00 ± 0.00)	(−7.94 ± 0.79^c^)	(−12.80 ± 0.80^cd^)	(−17.60 ± 0.80^d^)	(−23.02 ± 0.79^d^)	(−25.99 ± 0.79^d^)

100 mg/mL	211.67 ± 3.33^aA^	188.33 ± 1.67^cdB^	175.00 ± 2.89^dC^	163.33 ± 1.67^dD^	151.67 ± 1.67^dE^	148.33 ± 1.67^dE^
	(0.00 ± 0.00)	(−10.32 ± 0.79^c^)	(−16.00 ± 1.39^d^)	(−21.60 ± 0.80^d^)	(−27.78 ± 0.79^e^)	(−29.92 ± 0.79^d^)

*Note:* Means and SEM that do not share a lowercase letter along the column, as well as means of percentage change in heart rate (within parenthesis) that does not share a letter along the column are, significantly different. Means that do not share an uppercase superscript letter along the row, are significantly different (*p* < 0.05).

**Table 5:** Ionotropic effects of *S. incanum* extracts on isolated rabbit's heart.

Treatment group	0 min	Height of force of contraction (mm)				
		1^st^	2^nd^	3^rd^	4^th^	5^th^
Baseline	2.93 ± 0.07^Aa^	3.00 ± 0.12^cA^	3.00 ± 0.00^dA^	3.00 ± 0.12^cA^	2.87 ± 0.13^cA^	2.93 ± 0.07^dA^
Acetylcholine	2.93 ± 0.07^aA^	2.73 ± 0.03^dAB^	2.65 ± 0.03^eB^	2.53 ± 0.03^dBC^	2.40 ± 0.03^dC^	2.38 ± 0.04^eC^
Adrenaline	2.93 ± 0.03^aD^	3.97 ± 0.03^aC^	4.13 ± 0.03^aBC^	4.30 ± 0.03^aB^	4.51 ± 0.04^aA^	4.70 ± 0.06^aA^
25 mg	2.90 ± 0.06^aE^	3.23 ± 0.03^bcD^	3.42 ± 0.04^cCD^	3.58 ± 0.04^bBC^	3.72 ± 0.04^bB^	4.00 ± 0.04^cA^
50 mg	2.97 ± 0.03^aE^	3.33 ± 0.03^bD^	3.62 ± 0.04^bC^	3.77 ± 0.04^bBC^	3.92 ± 0.04^bB^	4.15 ± 0.03^bcA^
100 mg	2.92 ± 0.06^aE^	3.48 ± 0.02^bD^	3.70 ± 0.03^bC^	3.83 ± 0.04^bBC^	3.97 ± 0.03^bB^	4.30 ± 0.03^cA^

*Note:* Means and SEM that do not share a lowercase superscript letter along the column are significantly different (*p* < 0.05). Means and SEM that do not share an uppercase superscript letter are significantly different.

**Table 6:** Qualitative phytochemical analysis of *Solanum incanum* extracts.

Secondary metabolites	*Solanum incanum*
Saponins	+
Alkaloids	+
Terpenoids	+
Flavonoids	+
Glycosides	+
Steroids	+
Phenolics	+

*Note:* + = presence; − = absence.

**Table 7:** Effects of *S. incanum* extracts on body weight in mice.

Days	Percentage change (body weights) (g)			
	Baseline	100 mg/kg	174 mg/kg	300 mg/kg
7^th^ day	7.88 ± 0.84^aD^	5.91 ± 1.07^aD^	6.09 ± 1.00^aD^	5.91 ± 1.00^aD^
14^th^ day	14.08 ± 1.04^aC^	15.12 ± 0.99^aC^	14.91 ± 1.06^aC^	16.12 ± 0.92^aC^
21^st^ day	28.12 ± 1.30^aB^	25.24 ± 0.39^abB^	25.44 ± 0.77^abB^	24.56 ± 0.74^bB^
28^th^	35.13 ± 0.58^aA^	33.58 ± 0.93^abA^	32.48 ± 1.11^abA^	31.34 ± 0.85^bA^
Total change	21.30 ± 0.50^a^	19.96 ± 0.38^a^	19.73 ± 0.42^a^	19.48 ± 0.63^a^

*Note:* Means and SEM that do not share a letter along the row are significantly different. Means and SEM that do not share an uppercase superscript letter along the column are significantly different (*p* < 0.05).

**Table 8:** Effect of *S. incanum* on hematological parameters in mice.

Treatment	Baseline	100 (mg/kg)	174 (mg/kg)	300 (mg/kg)
WBCs (∗10 ^ 9/L)	10.12 ± 0.59	9.83 ± 0.41	10.37 ± 0.52	11.23 ± 0.50
RBCs (∗10 ^ 12/L)	11.11 ± 0.27	10.65 ± 0.33	10.72 ± 0.33	10.57 ± 0.44
HB (g/dL)	16.10 ± 0.42	16.28 ± 0.54	15.90 ± 0.42	16.12 ± 0.37
HCT (%)	60.73 ± 1.43	60.63 ± 1.73	60.87 ± 1.50	60.00 ± 2.19
MCV (fL)	54.58 ± 0.76	56.98 ± 0.78	56.84 ± 0.77	56.78 ± 0.46
RDW (%)	19.79 ± 0.48	20.38 ± 0.66	20.34 ± 0.57	19.62 ± 0.46
PLT (∗10 ^ 9/L)	63.58 ± 1.21	63.80 ± 0.77	64.47 ± 1.81	62.45 ± 2.07
MPV (fL)	7.56 ± 0.26	7.74 ± 0.23	7.60 ± 0.14	7.54 ± 0.21
PDW (fL)	10.30 ± 0.41	9.93 ± 0.27	9.56 ± 0.57	9.06 ± 0.80
PCT (%)	0.68 ± 0.06	0.70 ± 0.06	0.81 ± 0.05	0.80 ± 0.02

*Note:* Values in the same row are not statistically significant (*p* > 0.05).

**Table 9:** Effect of *S. incanum* on liver function and renal function tests in mice.

Treatment	Baseline	100 (mg/kg)	174 (mg/kg)	300 (mg/kg)
ALP (U/L)	88.00 ± 0.83	89.20 ± 0.37	87.40 ± 0.92	87.20 ± 0.58
AST (U/L)	274.80 ± 1.30	275.80 ± 0.58	275.60 ± 0.81	278.80 ± 1.16
ALT (U/L)	117.80 ± 0.73	117.20 ± 0.73	117.40 ± 0.67	118.60 ± 0.74
TP (U/L)	6.70 ± 0.11	6.72 ± 0.05	6.82 ± 0.12	6.94 ± 0.10
ALB (U/L)	3.55 ± 0.11	3.48 ± 0.14	3.56 ± 0.03	3.66 ± 0.34
TBIL (U/L)	25.40 ± 0.51	26.20 ± 0.37	26.80 ± 0.49	27.20 ± 0.66
DBIL (U/L)	10.72 ± 0.18	10.80 ± 0.22	11.28 ± 0.26	11.12 ± 0.38
CREAT (μmol/L)	64.18 ± 0.90	66.22 ± 0.84	64.84 ± 0.23	63.28 ± 0.50
Urea (μmol/L)	524.20 ± 7.15	518.20 ± 1.74	517.40 ± 3.19	525.49 ± 6.40
Na^+^ (μmol/L)	143.20 ± 1.08	142.80 ± 0.58	142.80 ± 0.80	144.40 ± 0.60
K^+^ (μmol/L)	5.10 ± 0.34	4.68 ± 0.23	4.82 ± 0.03	5.18 ± 0.07
Cl^−^ (μmol/L)	105.80 ± 0.58	105.60 ± 0.40	105.20 ± 0.49	106.00 ± 0.44
Ca (μmol/L)	2.31 ± 0.09	2.17 ± 0.06	2.21 ± 0.03	2.56 ± 0.12
P (μmol/L)	1.45 ± 0.12	1.41 ± 0.11	1.67 ± 0.08	1.67 ± 0.10

*Note:* Values in the same row are not statistically significant (*p* > 0.05).

## Data Availability

The data that support the findings of this study are available from the corresponding author upon reasonable request.
